# (Hyper)graph Kernels over Simplicial Complexes

**DOI:** 10.3390/e22101155

**Published:** 2020-10-14

**Authors:** Alessio Martino, Antonello Rizzi

**Affiliations:** Department of Information Engineering, Electronics and Telecommunications, University of Rome “La Sapienza”, Via Eudossiana 18, 00184 Rome, Italy; antonello.rizzi@uniroma1.it

**Keywords:** hypergraphs, graph kernels, kernel methods, support vector machines, simplicial complexes, topological data analysis

## Abstract

Graph kernels are one of the mainstream approaches when dealing with measuring similarity between graphs, especially for pattern recognition and machine learning tasks. In turn, graphs gained a lot of attention due to their modeling capabilities for several real-world phenomena ranging from bioinformatics to social network analysis. However, the attention has been recently moved towards hypergraphs, generalization of plain graphs where multi-way relations (other than pairwise relations) can be considered. In this paper, four (hyper)graph kernels are proposed and their efficiency and effectiveness are compared in a twofold fashion. First, by inferring the simplicial complexes on the top of underlying graphs and by performing a comparison among 18 benchmark datasets against state-of-the-art approaches; second, by facing a real-world case study (i.e., metabolic pathways classification) where input data are natively represented by hypergraphs. With this work, we aim at fostering the extension of graph kernels towards hypergraphs and, more in general, bridging the gap between structural pattern recognition and the domain of hypergraphs.

## 1. Introduction

Graphs are powerful data structures able to capture semantic and topological information from data. For this reason, graphs are commonly used to model a plethora of real-world, possibly complex, systems [1]. Notable examples include biological systems and chemistry [2,3,4,5,6,7,8,9,10,11,12,13], social and collaboration networks [14], computer vision and image processing [15,16,17,18], natural language processing [19,20,21,22], and energy distribution networks [23].

The price to pay for their modeling capabilities relies on the computational complexity needed in order to compare two graphs [24], as graphs lie in non-metric spaces and thus lack algebraic structures by definition [1]. To this end, several techniques have been proposed in literature in order to quantify (dis)similarities between graphs. Herein, three mainstream approaches are sketched, along with their respective major techniques, referring the interested reader to reviews such as those in [1,25].

*Graph matching* techniques evaluate the similarity directly in the graphs domain, conversely to the other two following approaches. In this family, it is possible to find

- the isomorphism test (i.e., two graphs of the same size can be considered equal if they are isomorphic) or the subgraph isomorphism test (i.e., if two graphs have different sizes, it is possible to check whether the smaller graph is isomorphic to a “portion” of the bigger graph);
- partial matching, which overcomes the drawback of the two above methods by the evaluation of the maximum (or minimum) common subgraph or the maximal common subgraphs [26,27,28,29]. In fact, isomorphism (by definition) does not consider partial similarities: two graphs can be isomorphic, not isomorphic or contained one into the other; and
- inexact graph matching, which does not consider an “exact” match between graphs or their respective subgraphs. Rather, (dis)similarity between graphs is evaluated by means of a properly formulated cost function, the seminal example being the (graph) edit distance [30,31], which evaluates the minimum cost sequence of atomic operations (insertion, deletion and substitution) on nodes and edges in order to transform the two graphs into one another.

Building an *embedding space* consists in moving the pattern recognition problem from the graphs domain towards a possibly metric (Euclidean) space: instead of using the former, one can use the latter which can be equipped with algebraic structures. To this family belongs

- dissimilarity spaces [32], where each pattern is described by the pairwise dissimilarities with respect to all training data (or a properly-chosen subset [3,33,34]), and
- embedding via information granulation, where recurrent and meaningful entities called *information granules* (e.g., network motifs, graphlets, and the like) are extracted by the training data and each pattern is cast into an integer-valued vector (*symbolic histogram*) which counts the number of occurrences of each granule within the pattern itself [35,36,37,38,39,40].

In *kernel methods*, the feature space is implicitly defined by means of a properly defined positive definite kernel function which projects the (possibly structured) data into a (possibly infinite-dimensional) Hilbert space in which data points are more likely to be linearly separable [41,42,43], e.g., by a maximal margin classifier such as support vector machines [44].

The aim of this paper is the introduction of four hypergraph kernels defined over simplicial complexes. In fact, while plenty of kernels have been designed for working in the domain of (labeled) graphs, there is still lack of kernel methods for hypergraphs. More in general, this is true for structural pattern recognition techniques, with the work in [36] being the only paper whose aim is to define suitable embedding spaces for hypergraph classification purposes. The strengths of the proposed kernels are as follows: their evaluation is not iterative; if the underlying graph is available, they are parameter-free; and, for some of them, the evaluation can be easily vectorized. A potential drawback is that (in their current implementation, at least) they only work on fully node labeled graphs, where nodes belong to a categorical and finite set (e.g., strings). The proposed kernels are compared to state-of-the-art graph kernels both in terms of efficiency and effectiveness on 18 benchmark datasets for graph classification. Computational results show that they generally outperform state-of-the-art graph kernels in terms of effectiveness and they are robust with respect to the size of the dataset. Furthermore, their parameter-free peculiarity makes the training phase appealing despite some state-of-the-art graph kernels outperform the proposed kernels in terms of evaluation time.

The remainder of the paper is structured as follows. In Section 2, the current literature on graph kernels is reviewed. In Section 3, hypergraphs and simplicial complexes are introduced. In Section 4, the four proposed kernels are described. Section 5 addresses the effectiveness and efficiency of the proposed kernels by comparing against state-of-the-art graph kernels (Section 5.1) and by facing a real-world biological case study (Section 5.2) in which input data are natively described by hypergraphs and current graph kernels cannot be employed. The properties of the proposed kernels are discussed in Section 6 (positive semi-definiteness) and Section 7 (computational complexity). Finally, Section 8 draws some conclusions and future directions.

## 2. Related Works on Graph Kernels

Graph kernels can be divided into four big families [25]. *Model driven kernels*, for example, exploit generative models or transformative models. An example of generative model driven kernel is the Fisher kernel [45], which exploits hidden Markov models. Conversely, an example of transformative model driven kernel is the diffusion kernel [46], which exploits the heat equation in order to diffuse local information to neighbor nodes.

The largest family is composed by *syntax driven kernels*, which aim at analyzing trees, paths, cycles or subgraphs. Notable examples include random walk kernels [47,48], shortest path kernels [49], graphlet kernels [50], and the Weisfeiler–Lehman kernels [51,52]. The first three, respectively, take into consideration the number of random walks between two graphs via their product graph, the number of common shortest paths, and the number of common *k*-order graphlets. The fourth one, instead, computes the number of subtrees-walks shared between two graphs using the Weisfeiler–Lehman test of graph isomorphism.

Another state-of-the-art approach is given by *propagation kernels* [53], which are based on the spread of information across several graphs using early-stage distributions from propagation schemes (e.g., random walks) in order to model different types of node and edge labels. Such propagation schemes make propagation kernels suitable for dealing also with unlabeled and partially labeled graphs.

Finally, *deep graph kernels* [54] can be seen as an extension of the aforementioned syntax driven kernels, where a positive semi-definite matrix weights (encodes) the relationships between sub-structures (e.g., graphlets).

For more information about these four families and a more exhaustive list of graph kernels belonging to each family, the interest reader is referred to the very detailed survey [25].

Fewer works have been done by using *hypergraphs*: hypergraphs can be seen as generalization of “plain graphs” where *hyperedges* can connect two or more nodes. In other words, hypergraphs extend the modeling capabilities offered by graphs (Section 1) in cases where multi-way relations are of interest; indeed, graphs take into account only pairwise relations [55,56,57,58,59]. A straightforward example may regard a scientific collaboration network in which *n* authors co-authored a paper: if one has to model this scenario using a graph, then one might consider nodes as authors which are connected by n(n−1)/2 edges. This modeling, however, is ambiguous about whether the *n* authors co-authored a paper or each pair of authors co-authored a paper. Using hypergraphs, an hyperedge can link the *n* authors, with no ambiguities. A more biologically-oriented example include protein interaction networks, where nodes correspond to proteins and edges exist whether they interact. Again, this representation does not consider protein complexes [60].

To the best of our knowledge, the only attempt to bridge the gap between hypergraphs and kernel methods is described in [61], where the authors proposed edit distance-based hypergraphlet kernels for node and vertex classification and link prediction, e.g., in protein–protein interaction networks.

Conversely to all previous works, in this paper four hypergraph kernels based on simplicial complexes for classification problems are proposed, therefore we exploit the multi-scale organization of complex networks addressing whether this modeling can also suit the definition of efficient and/or effective kernel functions for hypergraph classification tasks and foster the extension of graph kernels towards hypergraphs.

## 3. A Primer on Simplicial Complexes

A graph G={V,E} is defined by a finite set of vertices V and a set of edges E⊆V×V. Conversely, a hypergraph HG={V,H} is identified by a finite set of vertices V and a set of hyperedges H, namely, non-empty subsets of V. The striking difference between graphs and hypergraphs lies in their respective sets E and H: in fact, whereas E collects edges which encode pairwise relationships between nodes, H collects hyperedges, whose cardinality can be greater than two. In order words, H is able to collect multi-way relationships among elements in V.

Depending on the nature of the input data, building the corresponding hypergraph can be straightforward. Let us consider the example of the co-authorship network from Section 2. Given a paper *p*, with its respective authors list $a_{1},\ldots ,a_{n}$, it is possible to add to the hypergraph $a_{1},\ldots ,a_{n}$ as nodes and an hyperedge linking them. Furthermore, if authors $a_{1},\ldots ,a_{n}$ co-authored more than one paper together, the hyperedge can encode also this semantic information (i.e., the hyperedge is weighted with the number of papers that authors $a_{1},\ldots ,a_{n}$ co-authored together). An additional example (see later Section 5.2) may regard the hypergraph representation of a metabolic network. A metabolic network models the chain of chemical reactions occurring within the cell of a given living organism. It is well known from basic chemistry that chemical reactions can involve more than two molecules, therefore the hypergraph corresponding to a given metabolic network sees the molecules involved in any chemical reaction as vertices, with hyperedges that link together groups of molecules involved in the same chemical reaction.

Several representations of hypergraphs are available in the literature [59,62,63]. In the following, let us focus on one of the most commonly used one: simplicial complexes. Before diving into a formal definition of simplicial complex, it is worth defining the *simplex* of order (dimension) *k* (also, *k*-simplex) as the convex hull composed by (k+1) points. For example, points, lines, triangles, and tetrahedrons are 0-dimensional, 1-dimensional, 2-dimensional, and 3-dimensional simplices, being composed by 1, 2, 3, and 4 points (vertices), respectively, but also higher-order analogs exist. Every non-empty subset of the (k+1) vertices of a *k*-simplex is a *face* of the simplex: a face is itself a simplex. Therefore, a *simplicial complex* S can be formally defined as a finite collection (set) of simplices having the following two properties,

1. if σ∈S, then every face of σ is also in S, and
2. if $\sigma_{1},\sigma_{2}\in S$, then $\sigma_{1}\cap \sigma_{2}$ is a face of both $\sigma_{1}$ and $\sigma_{2}$.

A *subcomplex* of S is a subset of S which is also a simplicial complex: an important subcomplex is the *k*-skeleton, namely, a simplicial complex with at most simplices of order *k*. As the edge itself is a simplex, a graph can also be seen as a 1-skeleton (or 1-dimensional simplicial complex—where the *dimension* of a simplicial complex is defined as the maximum order among its simplices), being composed only by edges (1-dimensional simplices) and, eventually, nodes (0-dimensional simplices).

Several types of simplicial complexes exist and the choice mostly depends on their computational complexity and the cardinality of the starting data [64,65,66]. Simplicial complexes are widely used in the novel field of Topological Data Analysis in order to analyze a set of data by means of tools derived from topology. Therefore, conversely to the previous two examples (i.e., co-authorship network and metabolic network), in which the relationships between elements were perfectly described (i.e., well-known authors—paper and molecules—reaction information), usually data may come as a *point cloud*. A point cloud is defined as a finite set of points lying in some (possibly metric) space equipped with a notion of similarity. In this scenario, there are no a priori information about the connectivity among data points and several simplicial complexes can be employed in order to infer the simplicial structure and highlight the topological organization of the underlying data points. Notable examples include the Alpha complex and the Vietoris–Rips complex. The former relies on performing the Delaunay triangulation and then building the restricted Voronoi regions by intersecting (for each data point) an α-radius ball centered to it with its Voronoi region. The nerve of the set of all restricted regions is the Alpha complex at scale α. The latter is more graph-oriented and is defined as follows; a set of *k* points forms a (k−1)-dimensional simplex to be included in the Vietoris–Rips complex if all pairwise distances between points are less than or equal to a user-defined threshold ϵ. An efficient two-step procedure in order to compute the Vietoris–Rips complex has been proposed in [67]:

1. define the Vietoris–Rips neighborhood graph $G_{\mathrm{VR}}$ by scoring edges between any two vertices if their distance is less than or equal to ϵ and then
2. evaluate the flag complex of $G_{\mathrm{VR}}$.

In turn, the flag complex (also, clique complex) is the simplicial complex whose simplices are the maximal cliques in the underlying graph (1-skeleton). In other words, the clique complex is the topological space in which each *k*-vertex clique is represented by a (k−1)-simplex. Despite the minimalistic definition, it is straightforward to demonstrate that the flag complex is a valid simplicial complex: every maximal clique is a clique that cannot be made any larger and since every subset of a clique is also a clique (just like a face of a simplex is a simplex itself), then the closure under inclusion (i.e., closure under taking subsets) is automatically satisfied.

We stress the flag complex because of the following observation: simplicial complexes like the Vietoris–Rips complex strictly depend on a scale parameter (i.e., ϵ) which somewhat determines the resolution of the simplicial complex (i.e., the resolution at which data have been observed). Usually, by means of techniques such as persistence one finds a suitable value for ϵ [5]. However, if the underlying graph is already available, there is no need to build the neighborhood graph and find a suitable value for ϵ in order to build the Vietoris–Rips complex. Therefore, one can directly use the flag complex as it encodes the same information as the underlying graph, but it additionally completes a topological object with its fullest possible simplicial structure, being it a canonical polyadic extension of existing networks (1-skeletons) [55].

## 4. Proposed Hypergraph Kernels

From Section 3 it is clear that a simplex can be identified by considering the set of corresponding vertices. For example, when each vertex is just identified by a unique progressive number, it is impossible to match two simplicial complexes (e.g., determine how many/which simplices they have in common) and node labels play an important role: a simplex can be represented by the set of labels belonging to the corresponding vertices. Thanks to the node labels, the matching procedure is straightforward and can be done in an exact manner: two simplices (possibly belonging to different simplicial complexes) are equal if they have the same order and they share the same node labels. This observation is at the basis of all of the four kernels herein described.

### 4.1. The Histogram Cosine Kernel

The Histogram Cosine Kernel (HCK) is loosely based on the symbolic histogram technique. According to the latter, a given structured pattern (be it a graph, a sequence, and so on) can be explicitly embedded towards a Euclidean space by counting the number of recurrent and/or meaningful substructures (subgraphs, subsequences, and so on) properly extracted from the training data and classification can be performed in the Euclidean space spanned by these histogram vectors (for further details, the interested reader is referred to works such as in [1,35,39,68] and references therein). HCK is also inspired by the Graphlet Sampling kernel [50] and the Vertex Histogram kernel [69]. As the kernel evaluation relies on pairwise matching between patterns, the pivotal substructures to be considered for the symbolic histograms evaluation are the unique simplices between the two simplicial complexes to be matched. Specifically, let $S_{i}$ and $S_{j}$ be two simplicial complexes and let $A=S_{i}\cup S_{j}$ be the set of unique simplices belonging to either $S_{i}$ or $S_{j}$. Then, a given simplicial complex, say S, can be cast into a vector, say $f\in N^{\left|A\right|}$, where

(1) $$f_{k}=\mathrm{count}\left(A_{k},S\right),\;\forall k=1,\ldots ,\left|A\right|$$

where count(a,b) is a function that counts the number of occurrences of *a* in *b* and $A_{k}$ denotes the *k*-th element in A.

Therefore, thanks to Equation (1), $S_{i}\to f^{\left(i\right)}$ and $S_{j}\to f^{\left(j\right)}$, and HCK has the form

(2) $$K_{\mathrm{HC}}\left(S_{i},S_{j}\right)=\langle f^{\left(i\right)},f^{\left(j\right)}\rangle ,$$

where 〈·,·〉 denotes the plain dot product. However, HCK defined as in Equation (2) can be skewed by the different number of simplices within each simplicial complex. To this end, the following normalization is adopted,

(3) $$K_{\mathrm{HC}}\left(S_{i},S_{j}\right)=\frac{\langle f^{\left(i\right)},f^{\left(j\right)}\rangle }{\sqrt{\langle f^{\left(i\right)},f^{\left(i\right)}\rangle \cdot \langle f^{\left(j\right)},f^{\left(j\right)}\rangle }}.$$

Thanks to the product property of square roots, the latter can be written as

(4) $$K_{\mathrm{HC}}\left(S_{i},S_{j}\right)=\frac{\langle f^{\left(i\right)},f^{\left(j\right)}\rangle }{\sqrt{\langle f^{\left(i\right)},f^{\left(i\right)}\rangle }\cdot \sqrt{\langle f^{\left(j\right)},f^{\left(j\right)}\rangle }},$$

therefore collapsing into the cosine similarity between the vector representation (i.e., histogram) of the two hypergraphs, hence the name of the kernel.

### 4.2. The Weighted Jaccard Kernel

From Section 3 it is clear that simplicial complexes are sets. A straightforward idea is to use a similarity measure between sets as the core of the kernel. The seminal example is the Jaccard similarity, defined as the ratio between the size of the intersection divided by the size of the union between two sets. Since a simplex can be identified by the set of labels associated to each node, different simplices within the same simplicial complex can share the same node labels. This means that simplicial complexes are de facto multisets and the Jaccard similarity as previously defined loses its effectiveness and needs to be extended towards multisets. If $S_{i}$ and $S_{j}$ are two simplicial complexes to be matched, then their respective multiset representations read as

$$S_{i}=\left\{s_{1}^{\mu_{i}\left(s_{1}\right)},\ldots ,s_{n_{i}}^{\mu_{i}\left(s_{n_{i}}\right)}\right\}\;S_{j}=\left\{s_{1}^{\mu_{j}\left(s_{1}\right)},\ldots ,s_{n_{j}}^{\mu_{j}\left(s_{n_{j}}\right)}\right\}$$

where $n_{i}$ and $n_{j}$ denote the number of simplices in $S_{i}$ and $S_{j}$, respectively, and $\mu_{i}:S_{i}\to N_{\geq 0}$, $\mu_{j}:S_{j}\to N_{\geq 0}$ are the two multiplicity functions for $S_{i}$ and $S_{j}$. The latter is a function from the multiset (i.e., simplicial complex) towards the non-negative integers that returns the multiplicity (i.e., the number of occurrences) of an element (i.e., simplex *s*) within the multiset, with the caveat that if an element does not exist in the multiset, its multiplicity is 0. Let us now define the universe U from which the support of the two simplicial complexes are drawn

(5) $$U=\underset{\mathrm{support}\;\mathrm{of}\;S_{i}}{\underbrace{\left\{s\in S_{i}|\mu_{i}\left(s\right)>0\right\}}}\cup \underset{\mathrm{support}\;\mathrm{of}\;S_{j}}{\underbrace{\left\{s\in S_{j}|\mu_{j}\left(s\right)>0\right\}}}$$

Alike to the Jaccard similarity, the Weighted Jaccard Kernel is defined as the ratio between intersection and union of the two simplicial complexes that, being multisets, generalizes as

(6) $$K_{\mathrm{WJ}}\left(S_{i},S_{j}\right)=\frac{\min(\mu_{i}\left(s\right),\mu_{j}\left(s\right))}{\max(\mu_{i}\left(s\right),\mu_{j}\left(s\right))}\;\forall s\in U.$$

### 4.3. The Edit Kernel

The Edit Kernel (EK) aims at measuring the similarity between two simplicial complexes according to the number of simplices to be inserted, removed and/or substituted in order to transform the two simplicial complexes into one another. Let $e(S_{i},S_{j})$ be an edit distance (Levenshtein-like) with unitary weights. The first step is to convert the distance measure into a (possibly normalized) similarity measure. In [70], it has been demonstrated that

(7) $$\bar{e}\left(S_{i},S_{j}\right)=\frac{2\cdot e(S_{i},S_{j})}{|S_{i}\left|+\right|S_{j}|+e\left(S_{i},S_{j}\right)}$$

is a normalized edit distance in range [0,1] which satisfies the properties of a metric. Furthermore, in [71], it has been shown that if *d* is a normalized metric distance, then 1−d is a normalized metric similarity. Therefore, EK has the form

(8) $$K_{E}\left(S_{i},S_{j}\right)=1-\bar{e}\left(S_{i},S_{j}\right).$$

It is noteworthy that edit distances/similarities are sensitive to the order of the input sequences. In order to ease the matching procedure, within each simplicial complex, simplices are jointly sorted both lexicographically (i.e., according to the node labels) and according to their orders.

### 4.4. The Stratified Edit Kernel

The Stratified Edit Kernel (SEK) takes into account the following issue with EK: the latter can be skewed if the two simplicial complexes have a high variety of simplices per order. Let K be the set of different orders amongst simplices in the two simplicial complexes to be matched, then the SEK is defined as

(9) $$K_{\mathrm{SE}}\left(S_{i},S_{j}\right)=\frac{1}{\left|K\right|}\sum_{k\in K}1-\bar{e}\left(S_{i}^{\left(k\right)},S_{j}^{\left(k\right)}\right)$$

where $S^{\left(k\right)}$ denotes the subset of *k*-simplices in the simplicial complex S. The stratification allows to treat independently subsets of simplices having the same order; nonetheless, simplices are sorted lexicographically within each subset.

## 5. Tests and Results

### 5.1. On Benchmark Datasets

In order to investigate both the effectiveness and the efficiency of the proposed kernels, 18 datasets for graph classification have been considered. Details on the considered datasets (e.g., average number of nodes, average number of edges, number of samples, nodes and edges attributes), along with download links, can be found in [72]. No preprocessing has been performed on such data.

The four proposed kernels (HCK, WJK, EK and SEK) have been benchmarked against nine state-of-the-art graph kernels, listed below along with their respective parameters (and admissible values) to be tuned:

- Graphlet Sampling (GS) [50]. Parameters: the dimension of the graphlets k∈{3,4,5}
- Neighborhood Hash (NH) [73]. Parameters: number of hashes R∈{2,3,4,5} and the byte size of the hashes $2^{b}$ where b∈{2,3,4}. Other settings: simple hash type
- ODD-Sth (OS) [74]. Parameters: maximum single DAG height h∈{1,2,…,8}
- Propagation Kernel (PK) [53]. Parameters: number of iterations t∈{1,2,…,10} and LSH width $w\in [10^{-8},10^{-1}]$. Other settings: total variation as distance metric for LSH, base kernel set as the linear kernel
- Pyramid Match (PM) [75]. Parameters: pyramid histogram level L∈{2,3,4,5} and hypercube dimension d∈{2,…,10}.
- Random Walk (RW) [76]. Parameters: decay factor λ∈(0,0.5)
- SVM Theta (ST) [77]. Parameters: maximum size of the vertex set of sampled subgraphs M∈{2,3,…,10} (minimum size m=2) and number of samples s∈{20,21,…,100}. Other settings: product metric between theta numbers
- Weisfeiler–Lehman Subtree Kernel (WL) [52]. Parameters: number of iterations t∈{1,2,…,10}.
- Weisfeiler–Lehman Shortest Path Kernel (WLSP) [49,52]. Parameters: number of iterations t∈{1,2,…,10}. Other settings: shortest path matrices evaluated thanks to the Dijkstra algorithm.

As discussed in Section 3, the proposed four kernels are parameter-free (the flag complex can easily be evaluated using the Bron–Kerbosch algorithm [78,79]), whereas the same is not true for the nine competitors. A ν-SVM [80] has been used in order to perform classification. The regularization term ν∈(0,1], along with kernel parameters (if any) are learned via 10-fold cross-validation, driven by random-search [81]. For all datasets except FIRSTMM_DB, due to heavy unbalancing between number of splits and labels’ distribution, the cross-validation procedure has been stratified according to the ground-truth labels.

The four proposed kernels are implemented in Python, using NumPy [82] and NetworkX [83] as external dependencies, whereas the nine competitors belong to the GraKel library [84]. Finally, the ν-SVM implementation from the Scikit-Learn library [85] has been used for classification. The hardware setup includes a Linux CentOS 7 workstation equipped with two hyperthreaded 14-core Intel^®^ Xeon^®^ Gold 5120 CPU @ 2.20 GHz with 192 GB of RAM. Multithreaded parallelism has been exploited in order to speed up the optimization phase, where each thread processes the dataset by using a different set of parameters.

One final facet to stress before diving into the computational results lies on the graphs vs. hypergraphs duality: indeed, the datasets used for experiments are graph datasets (not hypergraphs), yet the proposed kernels are hypergraph kernels. The possibility to infer the simplicial structure from the underlying graph (e.g., thanks to the flag complex) has a crucial role in this regard, thanks to which one can infer hypergraphs starting from graphs, by adding additional information due to the definition of new possible *n*-ary relations [55,58]. This makes the four proposed kernels comparable with graph kernels (i.e., they both start from the same graphs). Notwithstanding that, it is safe to say that the four proposed kernels are indeed hypergraph kernels since they work on the top of the simplicial complexes that, in this case, have been obtained from the underlying 1-skeletons.

**Figure 1:**
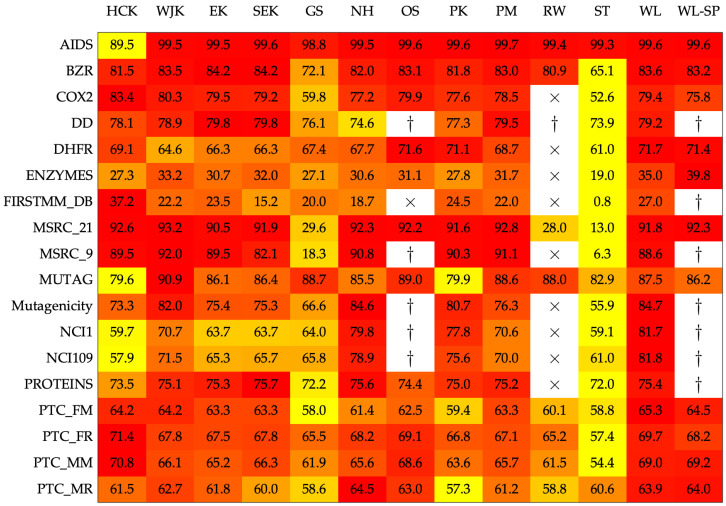
Average accuracy on the test set. The color scale has been normalized row-wise (i.e., for each dataset) from yellow (lower values) towards red (higher values, preferred). The times sign (×) indicates that the experiment has been aborted after passing a 24-h deadline. The dagger (†) indicates that the experiment went out-of-memory.

Figure 1 shows the results in terms of 10-fold cross-validation accuracy, a common practice in related research papers on graph kernels. Due to intrinsic randomness in both cross-validation and random search, each experiment has been repeated five times and the average value is shown. From Figure 1 it is possible to see that the four proposed kernels are competitive against the nine competitors. Furthermore, they are also very appealing for large datasets such as dd as no out-of-memory errors have been triggered and all executions terminated within the 24 h deadline (for details, see caption of Figure 1).

**Figure 2:**
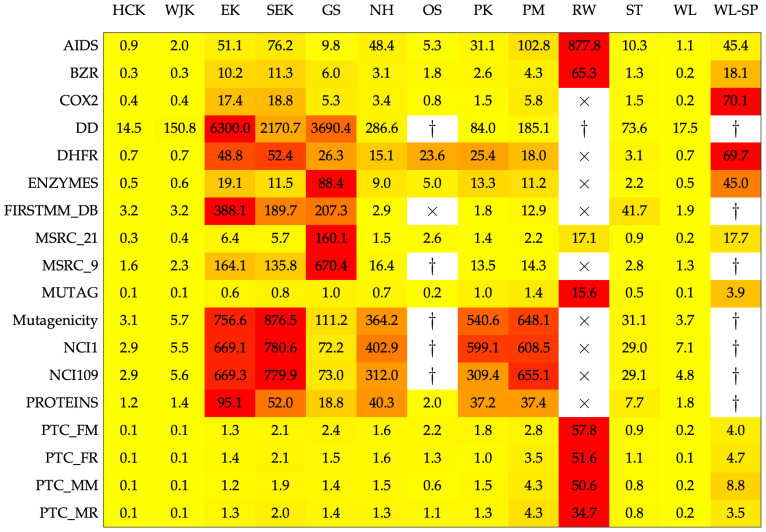
Average running times (in seconds) for evaluating the kernel matrix on the entire dataset. The color scale has been normalized row-wise (i.e., for each dataset) from yellow (lower values, preferred) towards red (higher values). The times sign (×) indicates that the experiment has been aborted after passing a 24-h deadline. The dagger (†) indicates that the experiment went out-of-memory. For the four proposed kernels, running times also include the simplicial complexes evaluation starting from the underlying graphs.

Conversely, Figure 2 shows the running times (in seconds) for evaluating the kernel matrix over the entire dataset. Wall-clock times refer to a single-threaded evaluation, no parallelism has been exploited. As the execution time is not deterministic due to spurious processes running in background, running times have been averaged across five evaluations of the kernel matrices. For the nine competitors, the best parameters returned by cross-validation have been used. From Figure 2 it is possible to see that RW is by far the slowest kernels to compute, followed by EK and SEK. The latter two kernels lack any vectorized statements, whereas the same is not true for HCK and WJK whose computational burden is competitive with current approaches. Notwithstanding the running times as such, it is worth stressing that the nine graph kernel competitors shall be evaluated several times in order to find a suitable set of parameters, whereas HCK, WJK, EK, and SEK can be evaluated only once.

### 5.2. A Biological Case Study: Analysis of Metabolic Pathways

In Section 5.1, a suite of 18 benchmark datasets for graph classification has been used in order to assess the performances of the proposed four kernels. Tests have been carried out against current approaches by inferring the simplicial complex from the underlying 1-skeletons in order to make them comparable. Here we present a biological case study in which entities are natively represented by hypergraphs and regards the classification of metabolic pathways. Metabolic pathways represent the chain of chemical reactions occurring in a cell and allow a straightforward network formalism [39,86,87]: vertices correspond to metabolites (product/substrate of a chemical reaction) and edges are scored whether there exists a chemical reaction transforming the two nodes into one another. Despite the network formalism has been extensively used in order to analyze metabolic pathways, some information is necessarily lost in the network formalization [88,89]. In fact, metabolic reactions often involve more than two reactants (e.g., A+B→C+D) and can be more conveniently represented by an hyperedge.

From the KEGG database, the metabolic pathways of 5299 organisms have been collected using its API in KGML format: KGML is the KEGG markup-like format in which each reaction in a given metabolic network is returned as a list of substrates and list of products. Therefore, the KEGG output can be interpreted as an hyperedge list, starting from which the native hypergraph modeling is straightforward. From the initial set of 5299 organisms, four classification problems are investigated by following the Linnaeus’ taxonomy at different scales:

- Problem 1 sees the entire set of 5299 organisms divided in two classes, according to their cellular architecture: Eukaryotes and Prokaryotes
- Problem 2 sees the entire set of 5299 organism divided in six classes, depending on their kingdom: Animals, Archaea, Bacteria, Fungi, Plants, and Protists
- Problem 3 sees a subset of 143 organisms (animals) divided in five classes, depending on their (Linnaeus’) class: Birds, Fishes, Insects, Mammals, and Reptiles
- Problem 4 sees a subset of 1456 organisms (bacteria) divided in seventeen classes, depending on their (Linnaeus’) class: Bacillus, Bifidobacterium, Burkholderia, Campylobacter, Chlamydia, Clostridium, Corynebacterium, Escherichia, Helicobacter, Lactobacillus, Mycobacterium, Mycoplasma, Pseudomonas, Salmonella, Staphylococcus, Streptococcus, and Streptomyces.

For additional information about the dataset, the interested reader is referred to the work in [39]. For these tests, a more thoughtful modeling has been employed in order also to address the generalization capabilities of the proposed kernels: for each of the four problems, the available patterns have been split in training set (50%), validation set (25%), and test set (25%) in a stratified manner in order to preserve labels’ distribution across the three splits. The best parameter $\nu^{★}$ is the one that maximizes the accuracy of the ν-SVM on the validation set after being trained on the training set. The best resulting model is finally evaluated on the test set. In order to take into account the intrinsic randomness in data splitting and hyperparameter tuning, for each problem, 10 experiments have been performed and results in terms of accuracy, specificity and sensitivity are shown in Table 1, Table 2 and Table 3, respectively. Clearly, the four proposed kernels show remarkable performances on the test set, regardless of the “resolution” at which the Linnaean taxonomy is investigated. Further discussion about the biological significance of being able to efficiently discriminate organisms according to their metabolic wiring can be found in [39,87].

## 6. On the Positive Definiteness of the Proposed Kernels

It is well known that a proper kernel function must satisfy the Mercer’s condition on positive (semi-)definiteness [90]. Herein, the positive (semi-)definiteness of the four proposed kernels is addressed. To this end, let us anticipate three theorems regarding well-known properties of positive (semi-)definite matrices.

**Theorem** **1.** *Let U and V be two positive semidefinite matrices, then their Hadamard product U⊙V (also known as element-wise product or point-wise product) is positive semidefinite. Furthermore, if U and V are positive definite, then U⊙V is positive definite.*

**Proof** **of** **Theorem** **1.** Given in [91]. See also in [42,92]. □

**Theorem** **2.** *Let $U_{1},\ldots ,U_{m}$ be m positive semidefinite matrices and let $a_{1},\ldots ,a_{m}$ be m non-negative scalars. Then, the matrix resulting from their linear combination $\sum_{i=1}^{k}a_{i}U_{i}$ is also positive semidefinite. Furthermore, if there exists a j∈{1,…,m} such that $a_{j}>0$ and $U_{j}$ is positive definite, then $\sum_{i=1}^{k}a_{i}U_{i}$ is positive definite.*

**Proof** **of** **Theorem** **2.** Given in [91]. See also in [42]. □

**Theorem** **3.** *Let U be a symmetric matrix (or Hermitian, in the complex case), then U is positive definite if all of its eigenvalues are positive. Furthermore, U is positive semi-definite if all of its eigenvalues are non-negative.*

**Proof** **of** **Theorem** **3.** Given in [91]. □

### 6.1. The Histogram Cosine Kernel

The core of the HCK is the pairwise evaluation of the cosine similarity between histograms. Therefore, showing that the cosine similarity is positive semi-definite suffices in order to show that HCK is a valid kernel. To this end, we give two equivalent proofs that exploit different properties of positive semi-definite matrices.

The first proof sees the cosine pairwise similarity matrix as written as the Hadamard product between two matrices, say N and D, respectively, defined as

(10) $$N_{i,j}=\langle x^{\left(i\right)},x^{\left(j\right)}\rangle$$

(11) $$D_{i,j}=\frac{1}{\sqrt{\langle x^{\left(i\right)},x^{\left(i\right)}\rangle }\cdot \sqrt{\langle x^{\left(j\right)},x^{\left(j\right)}\rangle }}$$

The former is the Gram matrix between histograms, therefore it trivially satisfies Mercer’s condition [91]. The latter has been shown to be positive semi-definite in [92]. Therefore, thanks to Theorem 1, HCK is a valid kernel.

The second proof involves a matrix, say X, with patterns (i.e., histograms) organized as rows, that is

(12) $$X=\left[\begin{array}{c} x^{\left(1\right)}\\ \vdots \\ x^{\left(N\right)} \end{array}\right]$$

Let us now normalize each row according to the $\ell_{2}$ norm of the row itself, that is

(13) $$\bar{X}=\left[\begin{array}{c} x^{\left(1\right)}\;/\;∥x^{\left(1\right)}∥\\ \vdots \\ x^{\left(N\right)}\;/\;∥x^{\left(N\right)}∥ \end{array}\right]$$

The cosine similarity matrix can dually be defined as $\bar{X}\cdot {\bar{X}}^{T}$, hence as the Gram matrix of $\bar{X}$, which trivially satisfies Mercer’s condition.

It is worth remarking that the space spanned by the symbolic histograms (cf. Equation (1)) can be equipped with any basic kernel for vector data: the choice of cosine similarity (i.e., normalized dot product) rather than, for example, polynomial or radial basis function stems from the work in [50,69].

### 6.2. The Weighted Jaccard Kernel

In order to show that WJK is a valid kernel, a vectorial representation of the kernel is analyzed. This also provides an efficient WJK evaluation procedure. It is well known that sets can be represented by binary vectors scoring 1 in position *i* if the *i*-th element exists in the set, and 0 otherwise. Similarly, multisets can be represented by integer-valued vectors which see in position *i* the value of the multiplicity function μ(·) for the *i*-th element: in other words, two simplicial complexes (say $S_{i}$ and $S_{j}$) can be transformed into vectorial collections (say $f^{\left(i\right)}$ and $f^{\left(j\right)}$) by following Equation (1). Under this light, the WJK (cf. Equation (6)) can be rewritten as

(14) $$K_{\mathrm{WJ}}\left(S_{i},S_{j}\right)=J_{W}\left(f^{\left(i\right)},f^{\left(j\right)}\right),$$

where

(15) $$J_{W}\left(a,b\right)=\frac{\sum_{i=1}^{n}\min\left(a_{i},b_{i}\right)}{\sum_{i=1}^{n}\max\left(a_{i},b_{i}\right)},\;\mathrm{for}\;a,b\in R^{n}$$

is known as weighted Jaccard similarity (or Ružička index). In order to begin with the proof, it is worth mentioning the following theorem.

**Theorem** **4.** *Let K(a,b) be a kernel function satisfying Mercer’s condition, then the following kernel is still valid,*

(16) $$\hat{K}\left(a,b\right)=\frac{K(a,b)}{K(a,a)+K(b,b)-K(a,b)}$$

**Proof** **of** **Theorem** **4.** Given in [93]. □

Equation (15) can be rewritten as follows,

(17) $$J_{W}\left(a,b\right)=\frac{\sum_{i=1}^{n}\min\left(a_{i},b_{i}\right)}{\sum_{i=1}^{n}a_{i}+\sum_{i=1}^{n}b_{i}-\sum_{i=1}^{n}\min\left(a_{i},b_{i}\right)},$$

and, by considering Equations (16) and (17), it can be rewritten as

(18) $$J_{W}\left(a,b\right)=\frac{\sum_{i=1}^{n}\min\left(a_{i},b_{i}\right)}{\sum_{i=1}^{n}\min\left(a_{i},a_{i}\right)+\sum_{i=1}^{n}\min\left(b_{i},b_{i}\right)-\sum_{i=1}^{n}\min\left(a_{i},b_{i}\right)}.$$

By comparing Equations (16) and (18) and by Theorem 4, it is clear that WJK is a valid kernel if $K\left(a,b\right)=\sum_{i=1}^{n}\min\left(a_{i},b_{i}\right)$ is a valid kernel as well. In order to demonstrate the latter, it is worth mentioning the following lemma.

**Lemma** **1.** *Let $\left\{a_{1},\ldots ,a_{m}\right\}$ be a finite set of real-valued numbers, then the matrix $M\in R^{m\times m}$ defined as*

(19) $$M_{i,j}=\min\left(a_{i},a_{j}\right)$$

*is a valid kernel matrix.*

**Proof** **of** **Lemma** **1.** Given in [92]. □

As WJK deals with real-valued vectors in $R^{n}$ and not with real-valued scalars as in the previous lemma, we shall extend the lemma to the former case. To this end, it is possible to build a series of *n* matrices, where each matrix (of the form as in Equation (19)) considers the pairwise minimum along a given dimension, say i=1,…,n, of the considered vectors and then perform the element-wise sum of such matrices:

(20) $$K\left(a,b\right)=M^{\left(1\right)}+\ldots +M^{\left(i\right)}+\ldots +M^{\left(n\right)}$$

Finally, Equation (20) can easily be shown to be a valid kernel matrix thanks to Theorem 2.

### 6.3. The Edit Kernel and Stratified Edit Kernel

Edit distances (similarities) are well known to lead to possibly indefinite kernels [29]. From Theorem 3, positive semi-definite matrices have non-negative eigenvalues. In order to quantify the goodness of the two proposed edit-based kernels, the negative eigenfraction (NEF) is investigated, defined as the relative mass of the negative eigenvalues [94]:

(21) $$NEF=\frac{\sum_{i:\lambda_{i}<0}\left|\lambda_{i}\right|}{\sum_{i}\left|\lambda_{i}\right|}.$$

**Figure 3:**
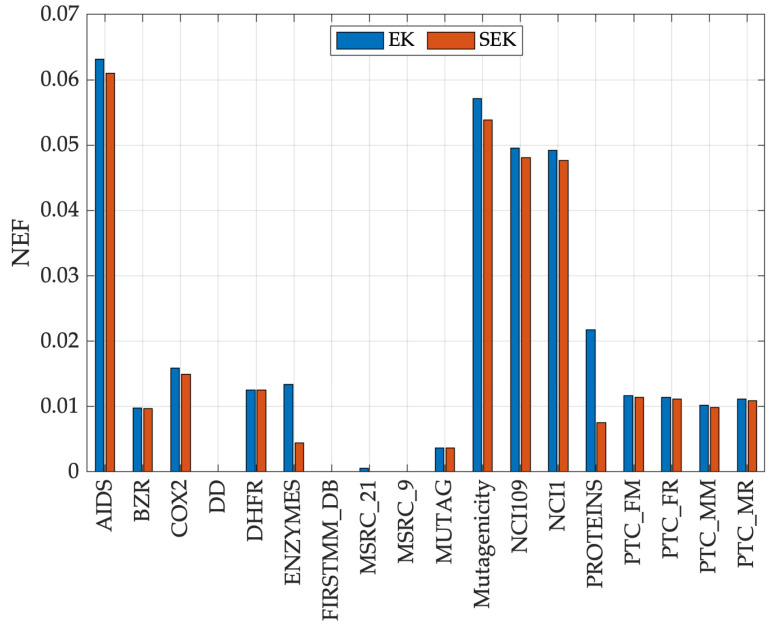
Negative Eigenfraction for the 18 tested datasets.

Clearly, NEF∈[0,1]: the closer to 0, the better. Figure 3 shows the NEF of the kernel matrices evaluated over the 18 datasets from Section 5 for EK and SEK. Both the proposed kernels have rather low NEF, the maximum being 0.0631 (dataset aids when using EK). Another interesting aspect is that SEK always outperforms EK in terms of NEF for all datasets (i.e., always less than or equal to). As they do not satisfy Mercer’s condition, it would be probably more suitable refer to those as *indefinite kernels*, following works such as [29,94,95], namely, “kernels” that despite their not being positive (semi-)definite perform reasonably good as confirmed, in our case, by competitive classification performances (Figure 1) and behavior remarkably close to a positive (semi-)definite kernel (Figure 3).

## 7. On the Computational Complexity of the Proposed Kernels

Each of the four proposed kernels works on a properly defined simplicial complex. Let us discuss the case of the clique complex used in this work: recall that the clique complex is the topological space in which each *k*-vertex clique is represented by a (k−1)-simplex. For an *n*-vertex graph, the maximum number of cliques in the worst-case goes like $O(3^{n/3})$ [96] and the Bron–Kerbosh algorithm matches this bound [97]. Therefore, the worst-case cardinality of the clique complex also follows this bound. For the sake of completeness, the same same holds for the Vietoris–Rips complex [67].

For any two simplicial complexes, say $S_{1}$ and $S_{2}$ with cardinality $n_{1}=\left|S_{1}\right|$ and $n_{2}=\left|S_{2}\right|$, the evaluation of HCK and WJK is linear with the order of the two simplicial complexes, with a worst-case of $O(n_{1}+n_{2})$, that is, when the two simplicial complexes have no simplices in common. For EK and SEK, as a Levenshtein-like edit distance [98,99] has been used, that is, instead of adding/substituting/deleting letters between two words, simplices has been added/substituted/deleted between two simplicial complexes, the computational complexity grows as $O(n_{1}\cdot n_{2})$.

## 8. Conclusions

In this paper, four (hyper)graph kernels have been proposed in order to exploit the native multi-scale organization in complex networks. All of the four kernels rely on simplicial complexes, one of the possible hypergraph representations and, specifically, on the flag complex of the underlying graph. What makes the hypergraph paradigm preferable (for some applications) over the “plain graph” modeling is that the former better captures the information in the data by taking into account multi-way relations, whereas the latter intrinsically takes into account only pairwise relations. The four kernels exploit simplicial complexes under different lights: for HCK, an explicit embedding towards a vector space is performed before evaluating the linear kernel; WJK relies on the set (or, better, multiset) structure of the simplicial complexes when equipped with semantic information on their respective nodes; EK and SEK measure the similarity in terms of edit operations defined on simplices.

All of the four kernels are easy to compute and rely on exact matching procedures among simplices given the categorical nature of the node labels. This is at the same time a strength and a weakness of the proposed kernels since edge labels and node attributes are not allowed. Future research endeavors can extend the proposed kernels to work with more complex node and/or edge labels.

An interesting aspect of the proposed kernels relies on them being parameter-free: if the underlying graph is available, its simplicial complex can be directly evaluated without any parameters to be tuned. This also holds if data natively comes as an hypergraph. This one-shot evaluation makes the training phase incredibly fast and appealing, as there is no need to evaluate multiple times the kernel matrix with different parameters and the only hyperparameter(s) to be tuned regard the classifier itself. Conversely, if the underlying graph is not available and data (nodes) come as a point cloud, the proposed kernels can still be employed, but they loose their parameter-free peculiarity: indeed, the flag complex cannot be directly used if the 1-skeleton is not natively available and one shall consider different simplicial complexes, such as the Vietoris–Rips complex or the Alpha complex, both of which strongly depend on a scale parameter (ϵ and α, respectively, cf. Section 3) which shall be tuned accordingly.

The four proposed kernels (HCK, WJK, EK, and SEK) have been benchmarked against nine state-of-the-art graph kernels (GS, NH, OS, PK, PM, RW, ST, WL, and WL-SP) on 18 open access datasets for graph classification. From this analysis, the following results emerged; the four kernels have competitive performances against current graph kernels, with HCK being slightly less accurate. Thanks to their parameter-free peculiarity, all of the proposed kernels have very fast training procedures. Furthermore they are also very appealing for large datasets, conversely to kernels such as OS, RW and WL-SP that went over the 24 h deadline or triggered out-of-memory errors. A second analysis regards the classification of metabolic pathways that, due to intrinsic molecular interactions, are conveniently represented by hypergraphs. Four classification problems are considered, following the Linnaeus’ taxonomy at different scales: from coarse-grained discrimination (e.g., eukaryotes vs. prokaryotes) to fine-grained discrimination (e.g., among bacteria). Regardless of the resolution at which organisms are classified, all of the four proposed kernels show remarkable performances: a clear sign that the four proposed kernels are able to capture the metabolic diversity across different layers of biological organization allowing, in turn, a consistent classification spanning at different scales.

By jointly considering performances and time required in order to evaluate the kernel matrix, WJK seems the most promising kernel among the proposed ones. HCK is featured by faster evaluation times, but its performances are slightly lower. For EK and SEK, their evaluation time is incredibly high due to lack of vectorization. However, future research avenues can investigate the possibility to use dedicated hardware in order to speed-up the edit distance evaluation between simplicial complexes, see, e.g., in [99]. Finally, WJK has also been demonstrated to be a valid kernel as the Mercer’s condition is satisfied.

## Figures and Tables

**Table 1:** Accuracy on the test set (average ± standard deviation) for the four metabolic pathways classification problems.

Kernel	Problem 1	Problem 2	Problem 3	Problem 4
HCK	99.98±0.05	99.95±0.05	98.89±1.84	99.89±0.18
WJK	100.0±0.0	99.91±0.07	98.61±1.86	99.89±0.25
EK	99.93±0.14	99.86±0.09	97.78±1.67	99.73±0.27
SEK	99.98±0.03	99.87±0.13	97.78±2.08	99.51±0.32

**Table 2:** Specificity on the test set (average ± standard deviation) for the four metabolic pathways classification problems.

Kernel	Problem 1	Problem 2	Problem 3	Problem 4
HCK	99.99±0.03	99.99±0.01	99.74±0.41	99.99±0.01
WJK	100.0±0.0	99.98±0.01	99.69±0.42	99.99±0.02
EK	99.96±0.07	99.98±0.02	99.49±0.38	99.98±0.02
SEK	99.99±0.02	99.98±0.02	99.52±0.44	99.97±0.02

**Table 3:** Sensitivity on the test set (average ± standard deviation) for the four metabolic pathways classification problems.

Kernel	Problem 1	Problem 2	Problem 3	Problem 4
HCK	99.99±0.03	99.82±0.28	99.4±1.28	99.91±0.15
WJK	100.0±0.0	99.67±0.51	97.2±3.45	99.89±0.27
EK	99.96±0.07	99.59±1.06	95.72±3.27	99.75±0.24
SEK	99.99±0.02	99.37±0.82	96.36±4.01	99.47±0.34

## Abbreviations

The following abbreviations are used in this manuscript.

EK	Edit Kernel
GS	Graphlet Sampling
HCK	Histogram Cosine Kernel
NEF	Negative EigenFraction
NH	Neighborhood Hash
OS	ODD-Sth
PK	Propagation Kernel
PM	Pyramid Match
RM	Random Walk
SEK	Stratified Edit Kernel
ST	SVM Theta
SVM	Support Vector Machine
WJK	Weighted Jaccard Kernel
WL	Weisfeiler–Lehman (Subtree Kernel)
WLSP	Weisfeiler–Lehman Shortest Path Kernel
